# Field Assessment of the Predation Risk - Food Availability Trade-Off in Crab Megalopae Settlement

**DOI:** 10.1371/journal.pone.0095335

**Published:** 2014-04-18

**Authors:** Sebastián Tapia-Lewin, Luis Miguel Pardo

**Affiliations:** Instituto de Ciencias Marinas y Limnológicas, Laboratorio Costero Calfuco, Facultad de Ciencias, Universidad Austral de Chile, Valdivia, Chile; Institute of Marine Research, Norway

## Abstract

Settlement is a key process for meroplanktonic organisms as it determines distribution of adult populations. Starvation and predation are two of the main mortality causes during this period; therefore, settlement tends to be optimized in microhabitats with high food availability and low predator density. Furthermore, brachyuran megalopae actively select favorable habitats for settlement, via chemical, visual and/or tactile cues. The main objective in this study was to assess the settlement of *Metacarcinus edwardsii* and *Cancer plebejus* under different combinations of food availability levels and predator presence. We determined, in the field, which factor is of greater relative importance when choosing a suitable microhabitat for settling. Passive larval collectors were deployed, crossing different scenarios of food availability and predator presence. We also explore if megalopae actively choose predator-free substrates in response to visual and/or chemical cues. We tested the response to combined visual and chemical cues and to each individually. Data was tested using a two-way factorial design ANOVA. In both species, food did not cause significant effect on settlement success, but predator presence did, therefore there was not trade-off in this case and megalopae respond strongly to predation risk by active aversion. Larvae of *M. edwardsii* responded to chemical and visual cues simultaneously, but there was no response to either cue by itself. Statistically, *C. plebejus* did not exhibit a differential response to cues, but reacted with a strong similar tendency as *M. edwardsii*. We concluded that crab megalopae actively select predator-free microhabitat, independently of food availability, using chemical and visual cues combined. The findings in this study highlight the great relevance of predation on the settlement process and recruitment of marine invertebrates with complex life cycles.

## Introduction

Settlement is a key process in the life cycle of meroplanktonic organisms as it determines the distribution of adult populations and community structure [1], [2]. This is mainly because settlers undergo high mortality rates as a consequence of their extreme vulnerability to a variety of factors during this period [3]. Starvation and predation are two important causes of mortality during settlement [4], [5]. Therefore, selective pressure promotes a pool of the morphological traits and larval behavioral strategies that could drive settlement in microhabitats with high availability of food and refuge from predators [6], [7]. As a consequence, in most marine meroplankton selection of settlement habitat is not a random choice. For example, coastal fish and crabs usually use a nursery ground, where competent larvae settle in abundance on structurally complex habitat, which provide a visual refugee from predators [8]. Also, preference for settlement in microhabitats with abundant organic matter has been also reported and direct or indirect signals of food availability can be a strong settlement cue [7], [9].

Chemical and physical cues have been well documented as the mechanism by which megalopae choose suitable habitats for settlement, reviewed in [10]–[12]. Historically, studies of this kind have been conducted mostly on sessile animals [6], [13], [14]. This is mainly because, in theory, mobile organisms do not need to be as selective, because they can actively move after settlement to more suitable areas (i.e. secondary dispersal), a process observed in several species of decapod brachyurans [15]–[17].

However, mobile crustacean species show the ability to delay metamorphosis in the absence of appropriate habitat cues, which indicates strong evidence for the importance of habitat quality for settlement [11]. This delay is temporary and frequently ends in spontaneous metamorphosis in the absence of stimuli (e.g. *Callinectes sapidus* [18], *Hemigrapsus sanguineus* [19], [20]). Studies about cues influence on megalopae behavior mainly test their response to positive cues, like adult habitat (biofilm, associated biota, substrates) and conspecific adult cues, the last being the most reported and consistent chemical cue for megalopae metamorphosis [12]. Few studies have assessed avoidance of potential settlement substrates, specifically how megalopae respond to microhabitat with potential predators, including conspecifics [21]–[24].

In decapods, two settlement patterns can be found in relation to conspecific presence; settlement induction or settlement avoidance. The first is common in gregarious species [25], [26] and associated with adult presence as indicator of habitat quality. The second is common in cannibalistic species, where competent larvae actively choose structurally heterogeneous and/or cryptic habitats that serve as refugee against juvenile cohorts [4], [27]. There are several reports of cannibalism, where megalopae and early post-settlers are actively preyed upon by older cohorts (*Carcinus maenas*: [28], [29]; *Neohelice granulata*: [30]; *Lithodes santolla*: [31]; *Acanthocylus* spp. [32]).

Thus, two main factors may control settlement at a micro-scale, the negative stimulus of predation risk (including cannibalism) and the positive stimulus of food availability. Although these factors have been recognized as key in the settlement process, there are few studies that assess a trade-off between them and their relative effect on settlement success in brachyurans [5], [33], [34].

*Metacarcinus edwardsii* (Bell, 1835) and *Cancer plebejus* (Poeppig, 1836) are two sympatric cancrid crabs, faced with intense fishery exploitation all along the Chilean coast, but populations are concentrated along the southern coast [35]. Previously, both species have been recorded recruiting in abundance in estuarine environments and larval supply to settlement habitats has been associated to tidal current and post-upwelling thermal fronts [36]–[37]. While the meso-scale pattern of settlement in estuaries is beginning to be understood, micro-scale processes remain largely unstudied.

Once these megalopae arrive in estuaries they actively choose complex substrates such as shell hash or algal tuft [36]–[38]. Early juvenile crabs seem not be significantly consumed by large predators [38], which therefore do not modify the general settlement patterns [36]. However, in these species, inter-cohort cannibalism or predation by juveniles of other crab species has not been evaluated.

This study aims to explore the micro-scale settlement pattern of two conspicuous and abundant crabs in an austral estuarine environment. First, we assess the relative importance (trade-off) of predation risk and food presence for these species on successful settlement. Then we determine if these megalopae respond to predator presence via chemical and/or visual cues in order to actively avoid unsuitable microhabitats for settlement.

## Materials and Methods

### Sampling Area

Field experiments were conducted in Corral Bay, at the mouth of the Valdivia-Tornagaleones estuary, located in southern Chile (39°50′53″ S, 73°23′44″W). This bay is characterized by a semidiurnal tide regime, with an average tidal height of 0.8 m, ranging from 0,53 m to 1,48 m [39]. The thermal and hyaline structure of the water column vary seasonally, from salt wedge in winter and spring to partially mixed during summer and autumn [40].

### Larval Collectors

In both experiments (section 2.3 and 2.4), settlement was assessed using 0.2 m^2^ passive benthic megalopae collectors [38]. A total of 545 collectors were deployed during recruitment seasons (November–December) between 2009 to 2011.These collectors offer the possibility of excluding or including large predators using an exclusion mesh (5 mm pore) which was placed on the top of the plastic trays (10 cm of height) and fixed by cables ties, which has no effect on larval settlement [38]. Collectors used in all treatments in this study, were (1) previously filled with 0.5 l of coarse sand, which is the preferred settlement habitat for these species [38]; (2) collectors were deployed on the estuarine bottom between 5–8 m depth; (3) exposed for 24 h, in order to include a complete tidal cycle; and (4) covered with the predator exclusion mesh.

### Relative Importance of Food and Predator Presence on Micro-scale Settlement

**Figure 1:**
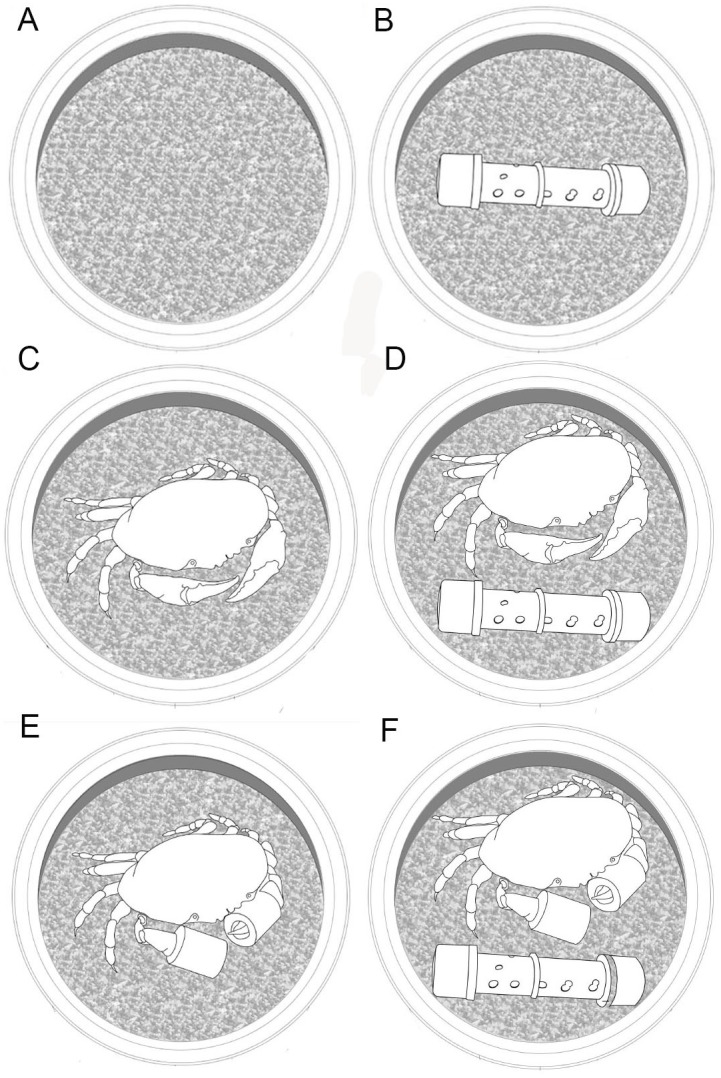
Experimental treatments to evaluate the trade-off of food availability and predator presence. Predators utilized were 1 year-old cohorts of *Metacarcinus edwardsii.* (Carapace Width  = 35±5 mm, not to scale). Exclusion mesh (non showed), which has no effect in settlement were placed in all treatments. Control and treatments were collectors with; A) only coarse sand, B) food provided, C) free predator, D) free predator with food provided, E) chelae-secured predator, F) chelae-secured predators with food provided.

This experiment was designed to determine the trade-off between predation and food availability on the settlement of both species. Variation in crab settlement was evaluated based on a full combination of predation presence and food availability levels. Predators were late instars of *M. edwardsii* (i.e. one year old cohorts) of 35±5 mm carapace width (CW). These instars feed upon megalopae and early instars of *M. edwardsii* and *C. plebejus* (Pardo LM, unpublished data). Food stimuli utilized consisted in 5 g doses of salmon food pellet. Doses were placed inside plastic PVC tubes (30 mm diameter), which were perforated and attached to bottom of the plastic tray of the collector (Fig. 1b, d, e). Salmon pellet is fish-based food and easily standardized by weight.

In order to test the attractiveness of salmon pellet to crabs, preliminarily settlement of crabs in collectors with pellet was compared with settlement collectors with more traditional bait (5 g of Mytilidae eviscerate) inside the PVC tubes. 7 trials were conducted, where 5 collectors with coarse sand and mesh exclusion were used per treatment in each trial (n total = 35). No significant differences were observed (F_1,35_ = 1,43, *p* = 0.29), so salmon food pellet was utilized as a food source in the experiments.

A factorial design was performed, with Trial, Predator and Food as mean factors. For the predation factor, three levels were considered (1) no predator, (2) free predators, and (3) chelae-secured predators (Fig. 1). In the chelae-secured predator level, chelae were bound with 5 mm diameter plastic housing. The efficiency of chelae-secured technique, to neutralize predatory capacity of juvenile crab was not experimentally tested (only observations in laboratory), therefore we assume a predatory reduction rather than an absence of predatory activity. Crabs that have completely lost their chelae can consume sediment and very small infaunal animals but they should have difficulty to catch very active prey, like megalopae. Thus, effects on megalopae settlement associated with predator presence were evaluated regardless of a normal predatory activity. Two food factor levels were included: (1) food provided and (2) without food (Fig. 1).

The experiment was conducted during November–December in 2009 and 2010. These months have the highest settlement rates for both species studied in the Valdivia estuary [36], [37]. Five trials (i.e. sampling days) were carried out each year, with 5 replicates per treatment in each trial (total n = 300). The controls were collectors with coarse sand but no predator or food (Fig. 1a). Data was analyzed with a 3-way full factorial ANOVA in the Statistica 7 statistical package. Predation and food were considered fixed factors, while trial was considered a random factor to include daily variation in settlement success in the model. Sampling years were pooled, as they did not show significant differences in a former full model.

### Role of Visual and Chemical Cues in Megalopae Settlement

**Figure 2:**
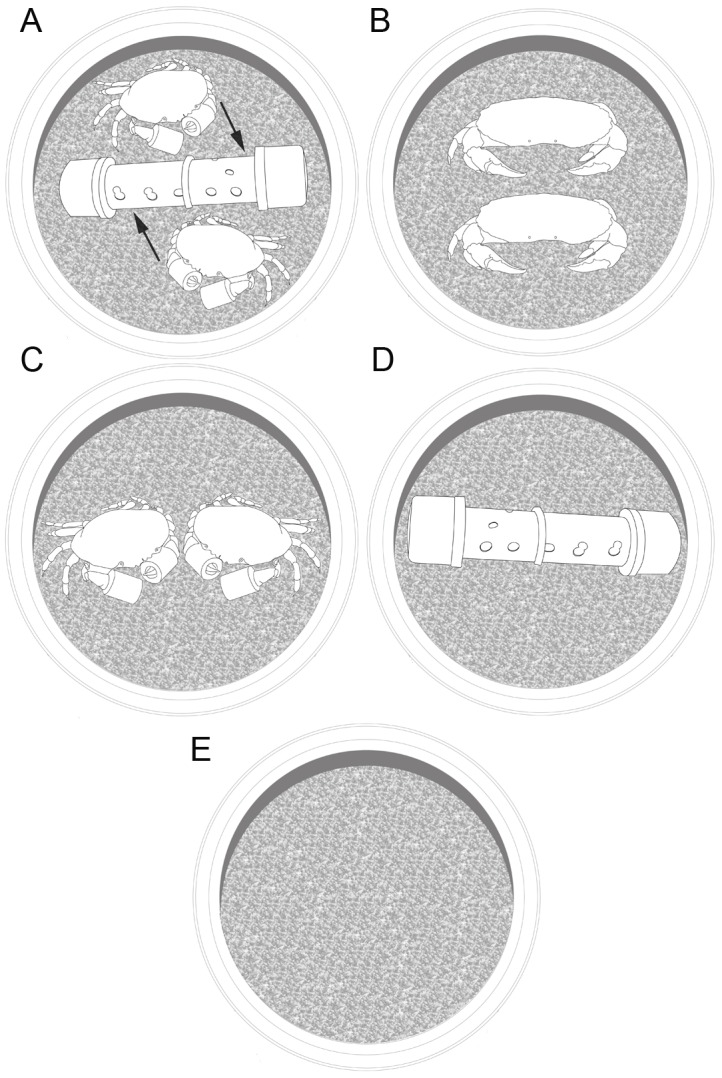
Experimental treatments to evaluate mechanisms of detection and avoidance of potential predators. Control and treatments were collectors with A) chelae-secured predators inside the PVC tubes to test response to chemical cues, B) plastic crab mimics to test visual cues; C) free chelae-secured predators to test visual and chemical cues combined; D) PVC tube control and E) collector control (only coarse sand).

This experiment was designed to determine if megalopae actively choose predator-free microhabitat via interpretation of visual and/or chemical cues of predators. Variation in crab settlement was evaluated on three treatments and two controls (Fig. 2). (1) Chemical odor from predators; consisting in predators (i.e. one-year old cohorts of *M. edwardsii*) hidden in perforated PCV plastic tubes (100 mm diameter). Hence, chemical cues could leave the tube, but the predator could not be seen by megalopae. Collectors were deployed with one perforated tube, containing two juvenile crabs with their chelae-secured (Fig. 2a). (2) Visual response to predators; in this treatment, two plastic juvenile crab mimics were placed inside the collectors, the size, color and form of mimics were similar to juvenile cancrid crabs (Fig. 2b). (3) Chemical and visual cues; the combined cues factor, were assessed by placing two chelae-secured predators in the collectors (Fig. 2c). (4) Procedure control used a collector with two perforated PVC plastic tubes, and (Fig. 2d) (5) another control collector with only with coarse sand, to detect crab settlement in absence of stimulus (Fig. 2e). Again, all collectors were covered with the exclusion mesh. Seven trials were carried out during November–December 2011, using seven collectors per trial per treatment (total n = 140). Data were analyzed with a 2-way ANOVA. Trial was set as random factor and treatment (cue type) as fixed factor.

### Sample Analysis and Statistical Considerations

Megalopae and first instars were identified via molecularly validated morphological traits, according to [41]. The response variable tested was settlement success expressed as settlers m^−^^2^ day^−^^1^. Variance homogeneity was tested with Cochrane’s test and normal distribution by a Kormogorov-Smirnov test before running the ANOVA. In all cases a logarithmic transformation (ln × +1) was required. Daily settlement of *M. edwardsii* fluctuates, with many days with of low megalopae abundance [37], given that the goal in this study is test the cues for settlement, days with average settlement below 1 settler m^−^^2^ day^−^^1^ or with more than a 75% occurrence of 0 settlers in collectors were not included in the analysis (3 trials were excluded). When statistically significant differences were found in ANOVAs, Fisher-LSD post-hoc test were performed to determine homogeneous groups. In the 3-way ANOVA, F-values were manually calculated according to [42] for 3-way ANOVA type III sum of squares.

### Ethics Statement

The research was performed in an open access marine area and no specific permissions are required to extract resources from these locations. Also, species involved in this research are not endangered or protected.

## Results

### Exploring the Potential Food Availability – predatory Risk Trade-off

**Figure 3:**
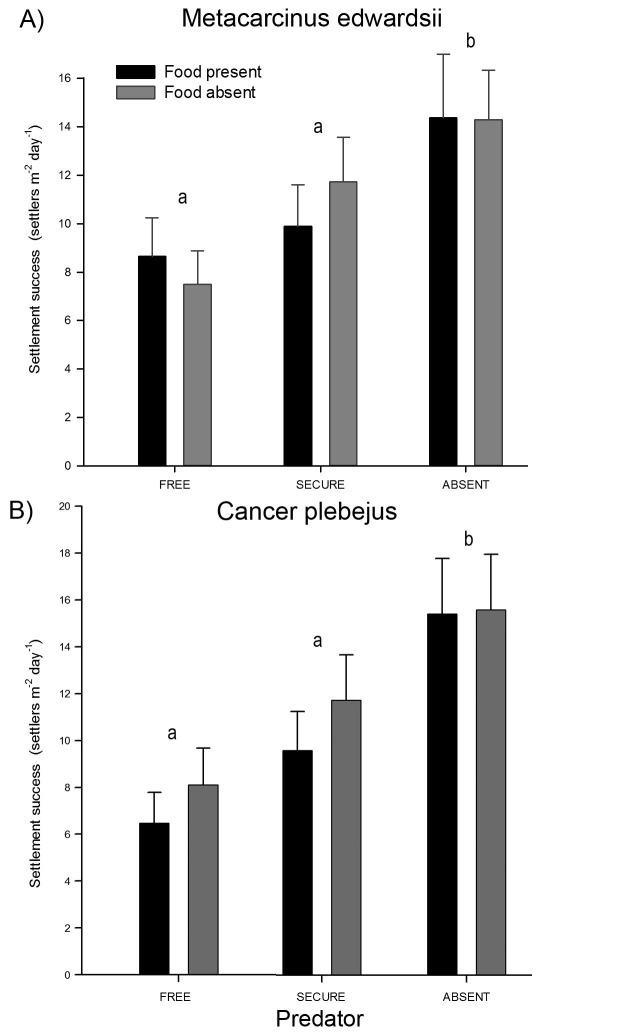
Mean settlement success based on predator presence and food availability scenarios for A) Metacarcinus edwardsii and B) Cancer plebejus megalopae. Different letters over bars indicate homogeneous groups within the different predation levels (food pooled) via Fisher-LSD post-hoc test. Error bars indicate standard deviation.

In *M. edwardsii* and *C. plebejus,* results showed that predator presence had greater relative importance than food availability on crab settlement success (Figure 3; Table 1). Interaction between these two factors was not statistically significant, indicating the absence of a trade- off (Table 1). Hence, micro-scale settlement success is mainly driven by predator detection by larvae, regardless of food availability. In both species, settlement success varied among trials, but this random factor did not interact with any other factor. Hence, the importance of predator presence is consistent even under fluctuating megalopae abundance.

**Table 1:** Three-way ANOVA for testing differences in settlement success of two Cancrid crabs on different scenarios of predator presence and food availability in different trials.

Source of variation	*Metacarcinus edwardsii*				*Cancer plebejus*			
	DF	MS	F	P-value	DF	MS	F	P-value
Trial (T)	9	13,029	32,842	0,276	**9**	**13,274**	**12,361**	**0,085**
**Predation (P)**	**2**	**4,835**	**4,635**	**0,024**	**2**	**8,642**	**13,927**	**≤0,001**
Food (F)	1	0,717	0,818	0,389	1	0,666	0,330	0,580
T×P	18	1,042	0,684	0,786	18	0,616	0,394	0,972
T×F	9	0,875	0,575	0,800	9	2,018	1,291	0,307
P×F	2	0,059	0,039	0,962	2	0,542	0,347	0,711
T×P×F	18	1,523	1,146	0,310	18	1,564	1,118	0,336
Error	221	1,329			223	1,399		

For *M. edwardsii*, free predators treatments showed only 57% of the settlement observed in treatments with no predators (LSD test, p≤0,001), and 75% compared to chelae-secured treatments. Treatments with chelae-secured predators showed a 25% reduction in settlement success when compared collectors with no predators (LSD test, p = 0,048). Again, food availability did not show a significant effect on settlement success (Table 1, Fig. 3a).

In the case of *Cancer plebejus*, treatments with free predators presented settlement success 33% lower than treatments with chelae-secured predator (LSD test, P = 0,062); and 50% of settlers was observed when compared to collectors with no predators. Collectors with chelae-secured predators showed 32% of the settlement success compared to treatments with no predators (Fig. 3b). Food availability did not have a statistically significant effect on settlement success (Table 1).

### Mechanisms of Megalopae Predator Avoidance

**Figure 4:**
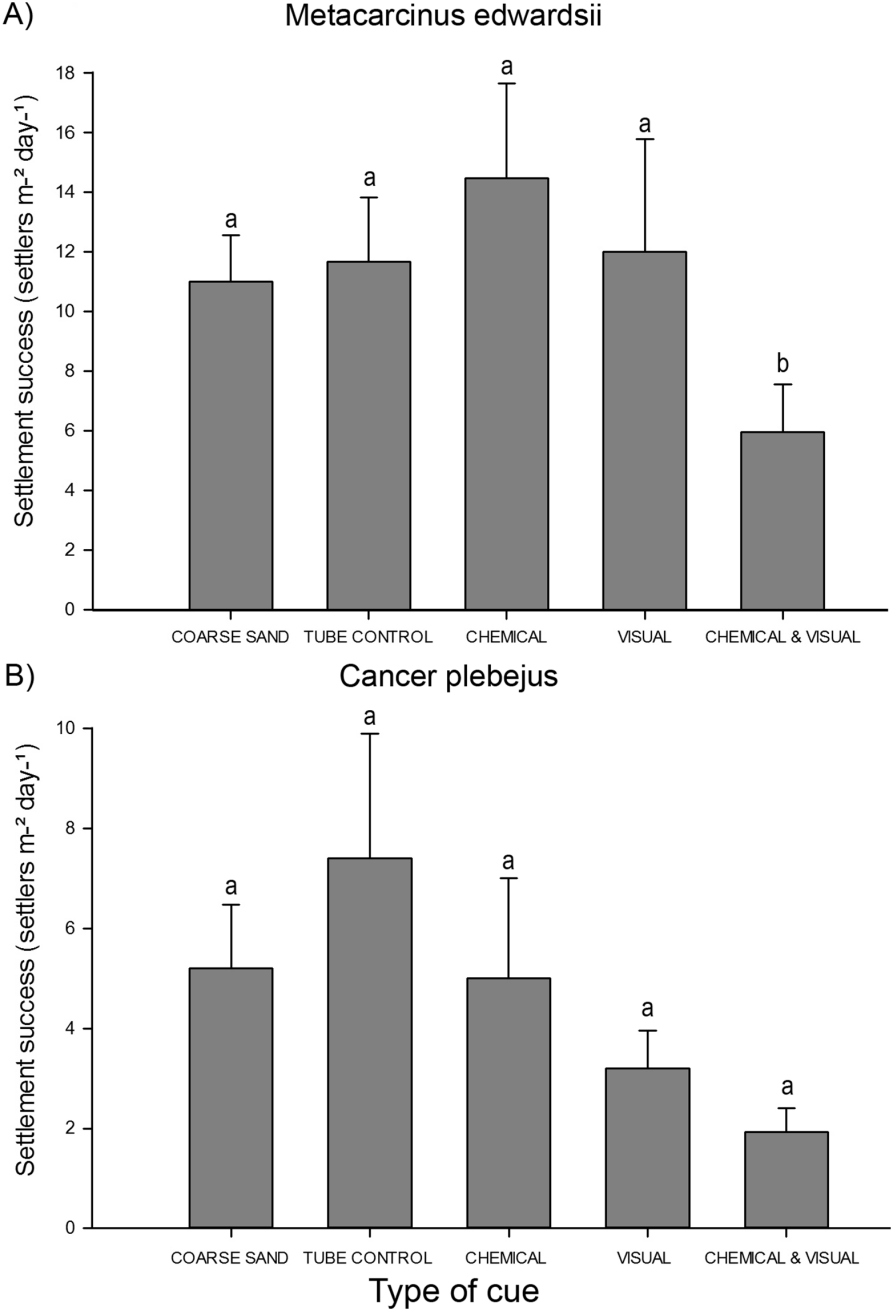
Mean settlement success based on response to chemical and visual cues from predators for A) *Metacarcinus edwardsii* and B) *Cancer plebejus*. Different letters over bars indicate homogeneous groups across treatments(*P*<0.05) via Fisher-LSD post-hoc test. Error bars indicate standard deviation.

*M. edwardsii* presented significant settlement succes differences with the presence of different predator cues (Table 2). Settlement success for treatments with chemical and visual cues combined was significantly lower than the rest of treatments (Table 2, Fig. 4a). Neither cue by itself was sufficient for megalopae to actively avoid collectors with predators. Visual and chemical cues combined inhibited settlement by approximately 50%, compared to other treatments (Fig. 3a).

**Table 2:** Two-way ANOVA for testing differences in settlement success of two Cancrid crabs responding to chemical and/or visual predatory cues.

Source of variation	*Metacarcinus edwardsii*				*Cancer plebejus*			
	DF	MS	F	P-value	DF	MS	F	P-value
**Trial (T)**	**3**	**11,332**	**12,834**	**≤0,001**	3	0,540	0,366	0,779
**Cue (C)**	**4**	**4,018**	**4,552**	**0,018**	4	2,745	1,859	0,182
TxC	12	0,880	0,693	0,755	12	1,479	1,147	0,331
Residual	111	1,270			108	1,289		

*Cancer plebejus* did not exhibit significant differences, despite showing a clear trend of decreased settlement when visual and chemical cues were combined (Fig. 4b). Daily differences were not observed in this trial, as opposed to *M. edwardsii* (Table 1, 2) when settlement per day was on average, 8 times lower. This finding highlights the importance of repeating experiments in time, especially when low settlement is observed, as effects may be masked by other conditions.

## Discussion

### Relative Importance of Predator and Food Presence

The direct effect of predation on crab settlers in field conditions is uncommonly studied [5.17.27], however competent larvae can undergo heavier predation pressure in the benthic environment, compared to the pelagic phase [43]. On the other hand, evidence of indirect predator control on decapod settlement has been frequently argued, suggesting that megalopae actively avoid predators by choosing heterogeneous substrates [38. 44]. This assertion is supported by findings from several studies, where lower mortality rates due to predation in these substrates have been recorded [17], [19], [38], [45]. For *Metacarcinus edwardsii* and *Cancer plebejus*, predator presence is a strong control on settlement by indirect mechanisms (triggering predator avoidance behavior).

Despite that food has been also been reported as an important metamorphosis and settlement inductor [7], [10], [46], in *M. edwardsii* and *C. plebejus,* food availability did not have a significant influence on settlement success as a mean factor or interacting with predation. Therefore, there is not an incentive to settle on substrates with high food availability but high predation risk, at least in the context of these experimental field conditions. Probably, the fact that the Valdivia-Tornagaleones fluvial system has a high organic matter load, which varies between 2 and 8% [47] and that both crabs can feed upon carrion, masks the relative importance of food to settlement. Future studies could analyze an organic matter gradient to estimate the effect on micro-scale habitat selection.

In a similar study [33], it was shown that the geometric complexity of the habitat was ecologically more important than food availability for settling megalopae of *Dyspanopues sayi*.Similar results were found for *Carcinus maenas* and *Paralithodes camtschaticus* [28], [48]. This indicates that the findings in this study, independent of field conditions, confirm the great relevance of predation as an indirect control on the settlement process of decapod megalopae.

### Predator Avoidance Mechanisms

*M. edwardsii* megalopae display effective avoidance behavior (i.e. choosing predator-free microhabitats) using a combination of visual and chemical cues. This could be a surprising finding, because (1) most positive stimulus for metamorphosis and settlement are chemical odors from adult substrate, aquatic vegetation, biofilms, co-specifics, estuarine water, related crab species, and potential prey; reviewed in [24], [11], [12], (2) other crab megalopae (*Callinectes sapidus)* actively avoid substrates containing only the odors from known predators [21]. However, that study did not test visual cues simultaneously, hence, it is uncertain if the response is exclusively chemical or if it may also be related to visual cues, and finally (3) in laboratory conditions, megalopae of *C. sapidus* respond to predatory chemical and visual cues, but separately [22]. These megalopae also preferred *Zostera marina* seaweed as a settlement substrate via chemical cues, but this behavior is reversed in the presence of the crab *Uca pugilator* and grass shrimp *Palaemonetes pugio* exudates, both predators of *C. sapidus* megalopae and first instars. *Hemigrapsus sanguineus* megalopae also respond separately to chemical and physical cues to settle in preferred habitats [19], but other crustacean species (*Xanthidae sp, Pachygrapsus planifrons, Lysiosquillina maculata* y *L. sulcata*) find their preferred habitat via visual cues alone and no response was observed when only chemical cues were present [49]. Thus, findings in this study show that megalopae visual capacity to perceive predators has been neglected as explicative mechanism for predator avoidance behavior.

Similar response to *M. edwardsii* only has been found studying the Florida stone crab (*Menippe mercenaria*) megalopae [50]. These larvae identify their preferred settlement substrate, the brown alga *Sargassum fluitans* via a combination of chemical and visual cues, but no response was elicited by either cue individually.

In aquatic organisms, olfactory (chemical) cues are a key source for early threat detection, being also the case for decapods [51], [52], [53], [54]. This is mainly due to the fact that chemical compounds in the water can effectively disperse across long distances [55]. Visual cues play an important role in detecting fine-scale predation menace [56]. Sensory cues may serve different roles, chemical cues can warn that a predator is nearby, and visual cues serve for a more accurate hazard evaluation [51], [52]. Despite low visibility in the sampling area, *M. edwardsii* megalopae respond to chemical and visual cues combined to avoid predators. This indicates that both are important, visual cues being possibly more relevant at short distances (cm) from the cue source [56].

Conspecific adult presence has been reported as the most consistent cue for larvae searching for suitable settlement habitats [11], [57], a response highly demonstrated in gregarious species (e.g. *Petrolisthes spp*: [58]). To our knowledge, this is the first study to assess, in the field, behavioral responses of megalopae to physical and chemical cues from juveniles of conspecific (in *M. edwardsii)* and closely related species (in *C. plebejus,* [59]), which are known to actively prey upon both species megalopae. This cannibalism and predation has been observed in other brachyurans (e.g. *Carcinus maenas*: [28], [29]; *Neohelice granulata:* [60]).

One of the reasons why literature reports that megalopae respond positively to cues conspecific adult presence (besides being an indicator of proper habitat for ontogenetic development) is because adults, by preying upon juveniles, facilitate megalopae settlement [30]. Cannibalism of megalopae is common among older cohort juveniles, but less common among adults, mainly due to the fact that megalopae are too small to be handled by adult chelae [27]. *Neohelice granulata* adults facilitate settlement of *Cytograpsus angulatus* and *Neohelice granulata* megalopae by actively preying upon *Neohelice granulata* juveniles, which prey upon megalopae of both species settlers [30].

Although settlement is not an irreversible process in brachyurans [28], according to our findings and literature review, generally megalopae are selective when choosing an appropriate microhabitat for a successful recruitment. *M. edwardsii* actively selects predator-free microhabitats, independent of food availability. This selection is made by interpretation of combined chemical and visual predatory cues, and no response was observed to each cue independently. Response is the same for *Cancer plebejus,* most likely due to the same mechanisms, although not yet experimentally proven, due to the very low settlement observed the year that question was approached. Future studies could assess megalopae response to conspecific/congeneric juvenile and adult cues (also suggested in other study [61]) and test if the response changes according to the ontogenetic state or phylogenetic distance used in essays. We also suggest studying the effect of an organic matter gradient on settlement.

## References

[1] Underwood A, Keough MJ (2001) Supply-side ecology the nature and consequences of variations in recruitment of intertidal organisms. In: Berteness M., Gaines S, & Hay M. (Eds.), Marine Community Ecology. Sinauer Associates Inc, Sunderland. 183–200.

[2] Gaines S, Roughgarden J (1985) Larval settlement rate: A leading determinant of structure in an ecological community of the marine intertidal zone. Proc Natl Acad Sci USA 82(11): 3707–3711. 16593571 10.1073/pnas.82.11.3707 PMC397856

[3] Caley MJ, Carr MH, Hixon MA, Hughes TP, Jones GP, et al (1996) Recruitment and the local dynamics of Open Marine Populations. Annu Rev Ecol Syst 26: 477–500.

[4] Fernandez M, Iribarne O, Armstrong D (1993) Habitat selection by Young-of-the-year Dungeness crab *Cancer magister* and predation risk in intertidal habitats. Mar Ecol Prog Ser 92: 171–177.

[5] Moksnes P-O (2002) The relative importance of habitat-specific settlement, predation and juvenile dispersal for distribution and abundance of young juvenile shore crabs *Carcinus maenas* . J Exp Mar Biol Ecol 271: 41–73.

[6] Keough MJ, Downes BJ (1982) Recruitment of marine invertebrates: the role of active larval choice and early mortality. Oecologia 54, 348–352. 10.1007/BF00380003 28309958

[7] Pirtle JL, Stoner AW (2010) Red king crab (*Paralithodes camtschaticus*) early post-settlement habitat choice: Structure, food, and ontogeny. J Exp Mar Biol Ecol 393: 130–137.

[8] Beck MW, Heck KL Jr, Able KW, Childers DL, Eggleston DB, et al (2001) The Identification, Conservation, and Management of Estuarine and Marine Nurseries for Fish and Invertebrates. BioScience 51: 633–641.

[9] Rodriguez SR, Ojeda FP, Inestrosa NC (1993) Settlement of Benthic Marine Invertebrates. Mar Ecol Prog Ser 97: 193–207.

[10] Forward RB Jr, Tankersley RA, Rittschof D (2001) Cues for metamorphosis of brachyuran crabs: an overview. Am Zool 41: 1108–1122.

[11] Gebauer P, Paschke K, Anger K (2003) Delayed metamorphosis in Decapod Crustaceans: evidence and consequences. Rev Chil Hist Nat 76: 169–175.

[12] Paul VJ, Ritson-Williams R, Sharp K (2011) Marine chemical ecology in benthic environments. Nat Prod Rep 28: 345–387. 21125086 10.1039/c0np00040j

[13] Stoner DS (1990) Recruitment of a tropical colonial ascidian: relative importance of pre-settlement vs post-settlement processes. Ecology 71: 1682–1690.

[14] Pechenik JA (1990) Delayed metamorphosis by larvae of benthic marine invertebrates: Does it occur? Is there a price to pay? Ophelia 32: 63–94.

[15] Thiel M, Zander A, Valdivia N, Baeza JA, Rueffler C (2003) Host fidelity of a symbiotic porcellanid crab: the importance of host characteristics. J Zool 261: 353–362.

[16] Underwood A (2004) Landing on one’s foot: small-scale topographic features of habitat and the dispersion of juvenile intertidal gastropods. Mar Ecol Prog Ser 268: 173–182.

[17] Pardo LM, Palma AT, Prieto C, Sepulveda P, Valdivia I, et al (2007) Processes regulating early post-settlement habitat use in a subtidal assemblage of brachyuran decapods. J Exp Mar Biol Ecol 344: 10–22.

[18] Wolcott DL, De Vries MC (1994) Offshore megalopae of *Callinectes sapidus*: depth of collection, molt stage and response to estuarine cues. Mar Ecol Prog Ser 109: 157–163.

[19] O’Connor NJ (2007) Stimulation of molting in megalopae of the Asian shore crab *Hemigrapsus sanguineus*: physical and chemical cues. Mar Ecol Prog Ser 352: 1–8.

[20] Steinberg MK, Krimsky LS, Epifanio CE (2008) Induction of metamorphosis in the Asian Shore Crab *Hemigrapsus sanguineus*: Effects of Biofilms and Substratum Texture. Estuaries Coast 31: 738–744.

[21] Welch JM, Rittschof D, Bullock TM, Forward RB Jr (1997) Effects of chemical cues on settlement behavior of blue crab *Callinectes sapidus* postlarvae. Mar Ecol Prog Ser 154: 143–153.

[22] Diaz H, Orihuela B, Forward RB Jr, Rittschof D (1999) Orientation of blue crab: *Caliinectes sapidus* (Rathbun), Megalopae: Responses to visual and chemical cues. J Exp Mar Biol Ecol 233: 25–40.

[23] Diaz H, Orihuela B, Forward RB Jr, Rittschof D (2001) Effect of chemical cues on visual orientation of juvenile blue crabs, *Callinectes sapidus* (Rathbun). J Mar Biol Ecol 266: 1–15.

[24] Forward RB Jr, Tankersley RA, Smith KA, Welch JM (2003) Effects of chemical cues on orientation of blue crab, *Callinectes sapidus* megalopae in flow: implications for location of nursery areas. Mar Bio. 142: 747–756.

[25] Jensen GC (1989) Gregarious settlement by megalopae of the porcelain crabs *Petrolisthes cinctipes* (Randall) and *P*. *eriomerus* Stimpson. J Exp Mar Biol Ecol 131: 223–231.

[26] Gebauer P, Walter I, Anger K (1998) Effects of substratum and conspecific adults on the metamorphosis of *Chasmagnathus granulata* (Dana) (Decapoda: Grapsidae) megalopae. J Exp Mar Biol Ecol 223: 185–198.

[27] Luppi TA, Spivak ED, Anger K (2001) Experimental studies on predation and cannibalism of the settlers of *Chasmagnathus granulata* and *Cyrtograpsus angulatus* (Brachyura: Grapsidae). J Exp Mar Biol Ecol 26: 29–48.

[28] Moksnes P-O, Hedvall O, Reinwald T (2003) Settlement behavior in shore crabs *Carcinus maenas*: why do postlarvae emigrate from nursery habitats? Mar Ecol Prog Ser 250: 215–230.

[29] Almeida MJ, Gonzalez-Gordillo JI, Flores AAV, Queiroga H (2012) Cannibalism, post settlement growth rate and size refuge in a recruitment-limited population of the shore crab *Carcinus maenas* . J Exp Mar Biol Ecol 410: 72–79.

[30] Méndez-Casariego A, Alberti J, Luppi T, Iribarne O (2009) Stage-dependent interactions between intertidal crabs: from facilitation to predation. J Mar Biol Ass UK 89(4): 781–788.

[31] Sotelano MP, Lovrich GA, Romero MC, Tapella F (2012) Cannibalism during intermolt period in early stages of the Southern King Crab *Lithodes santolla* (Molina 1872): effect of stage and predator-prey proportions. J Exp Mar Biol Ecol 411: 52–58.

[32] Januario SB, Navarrete SA (2013) Cannibalism and inter-specific predation in early stages of intertidal crab species that compete for refuges. J Exp Mar Biol Ecol 446: 36–44.

[33] Lindsey EL, Altieri AH, Witman JD (2006) Influence of biogenic habitat on the recruitment and distribution of a subtidal xanthid crab. Mar Ecol Prog Ser 306: 223–231.

[34] Krediet CJ, Donahue MJ (2009) Growth-mortality trade-offs along a depth gradient in *Cancer borealis* . J Exp Mar Biol Ecol 373(2): 133–139.

[35] SERNAPESCA (2011) Anuario de estadísticas pesqueras. Ministerio de Economía, Servicio Nacional de Pesca, Gobierno de Chile, 186.

[36] Pardo LM, Cardyn CS, Garcés-Vargas J (2012) Spatial variation in the environmental control of crab larval settlement in a micro-tidal austral estuary. Helgol Mar Res 66(3): 253–263.

[37] Pardo LM, Mora-Vasquéz P, Garcés-Vargas J (2012) Asentamiento diario de megalopas de jaibas del género *Cancer* en un estuario micromareal. Lat Am J Aquat Res 40(1): 142–152.

[38] Pardo LM, Cardyn CS, Mora-Vasquéz P, Wahle RA (2010) A new passive collector to assess settlement rates, substrate selection and predation pressure in decapod crustacean larvae. J Exp Mar Biol Ecol 393: 100–105.

[39] Pino M, Perillo GME, Santamarina P (1994) Residual fluxes in a cross section in the Valdivia River estuary, Chile. Est Coast Shelf Sci 38: 491–505.

[40] Garces-Vargas J, Ruiz M, Pardo LM, Nuñez S, Pérez-Santos I (2013) Caracterización hidrográfica del estuario del río Valdivia, centro-sur de Chile. Lat Am J Aquat Res 41(1): 113–125.

[41] Pardo LM, Ampuero D, Véliz D (2009) Using morphological and molecular tools to identify megalopae larvae collected in the field: the case of sympatric *Cancer* crabs. J Mar Biol Assoc UK 89(3): 481–490.

[42] Zar JH (1999) Biostatistical Analysis. New Jersey: Prentice Hall. 662p.

[43] Allen JD, McAlister JS (2007) Testing rates of planktonic versus benthic predation in the field. J Exp Mar Biol Ecol 347: 77–87.

[44] Moksnes P-O, Heck KL Jr (2006) Relative importance of habitat selection and predation for the distribution of blue crab megalopae and young juveniles. Mar Ecol Prog Ser 308: 165–181.

[45] Dittel A, Epifanio CE, Natunewicz C (1996) Predation on mud crab megalopae, *Panopeus herbstii* (H. Milne Edwards): Effect of habitat complexity, predator species and post-larval densities. J Exp Mar Biol Ecol 198(2): 191–202.

[46] Rodriguez RA, Epifanio CE (2000) Multiple cues for induction of metamorphosis in larvae of the common mud crab *Panopeus herbstii* . Mar Ecol Prog Ser 195: 221–229.

[47] Matamala TB (2011) Poliquetos asociados a los grampones de *Macrocystis pyrifera* dentro del estuario del Rio Valdivia: Variaciones en un gradiente estuarino. Thesis in Marine Biology, Escuela de Biología Marina, Facultad de Ciencias, Universidad Austral de Chile, 71.

[48] Stevens BG (2003) Settlement, substratum preference, and survival of red king crab *Paralithodes camtschaticus* (Tilesius, 1815) glaucothoe on natural substrata in the laboratory. J Exp Mar Biol Ecol 283, 63–78.

[49] Lecchini D (2011) Visual and chemical cues in habitat selection of sepioid larvae. Cr Soc Biol 334(12): 911–915. 10.1016/j.crvi.2011.08.003 22123093

[50] Krimsky LS, Epifanio CE (2008) Multiple cues from multiple habitats: Effect on metamorphosis of the Florida stone crab, *Menippe mercenaria* . J Exp Mar Biol Ecol 358: 178–184.

[51] Rittschof D, Tsai DW, Massey PG, Blanco L, Kueber GL Jr, et al (1992) Chemical mediation of behavior in hermit crabs: Alarm and aggregation cues. J Chem Ecol 18(7): 959–984. 24254141 10.1007/BF00980056

[52] El-Kareem TG (2012) Effects of visual and chemical cues on orientation behavior of the Red Sea hermit crab *Clibanarius signatus* . J Bas App Zool 65(2): 95–105.

[53] Hazlett BA, Mclay C (2005) Responses to predation risk: alternative strategies in the crab *Heterozius rotundifrons* . Anim Behav 69(4): 967–972.

[54] Chiussi R, Diaz H, Rittschof D, Forward R (2001) Orientation of the hermit crab *Clibanarius* antillensis: effects of chemical and visual cues. J Crust Biol 21(3): 593–605.

[55] Wiseden BD (2000) Olfactory assessment of predation in the aquatic environment. Philos Trans R Soc Lond 355: 1205–1208. 11079399 10.1098/rstb.2000.0668 PMC1692838

[56] Williamson JE, Gleeson C, Bell JE, Vaïtilingon D (2012) The role of visual and chemical cues in host detection by the symbiotic shrim *Gnathophylloides mineri.* . J Exp Mar Biol Ecol 414: 38–43.

[57] Anger K (2006) Contributions of larval biology to crustacean research: a review. Invertebr Reprod Dev 49(3): 175–205.

[58] Jensen GC (1991) Competency, settling behavior, and postsettlement aggregation by porcelain crab megalopae (Anomura: Porcellanidae). J Exp Mar Biol Ecol 153: 49–61.

[59] Schram FR, Ng PKL (2012) What is Cancer?. J Crust Biol 32(4): 665–672.

[60] Méndez-Casariego A, Alberti J, Luppi T, Daleo P, Iribarne O (2011) Habitat shifts and spatial distribution of the intertidal crab *Neohelice (Chasmagnathus) granulata* Dana. J Sea Res 66: 87–94.

[61] Diele K, Simith DJB (2007) Effects of substrata and conspecific odour on the metamorphosis of mangrove crab megalopae, *Ucides cordatus* (Ocypodidae). J Exp Mar Biol Ecol 348: 174–182.

